# The Effectiveness of a Web-Based Computer-Tailored Intervention on Workplace Sitting: A Randomized Controlled Trial

**DOI:** 10.2196/jmir.5266

**Published:** 2016-05-31

**Authors:** Katrien De Cocker, Ilse De Bourdeaudhuij, Greet Cardon, Corneel Vandelanotte

**Affiliations:** ^1^ Department of Movement and Sports Sciences Ghent University Ghent Belgium; ^2^ Research Foundation Flanders Brussels Belgium; ^3^ Physical Activity Research Group School for Human Health and Social Sciences Central Queensland University Rockhampton Australia

**Keywords:** sedentary behavior, computer tailoring, employees, activPAL, sitting time, randomized controlled trial

## Abstract

**Background:**

Effective interventions to influence workplace sitting are needed, as office-based workers demonstrate high levels of continued sitting, and sitting too much is associated with adverse health effects. Therefore, we developed a theory-driven, Web-based, interactive, computer-tailored intervention aimed at reducing and interrupting sitting at work.

**Objective:**

The objective of our study was to investigate the effects of this intervention on objectively measured sitting time, standing time, and breaks from sitting, as well as self-reported context-specific sitting among Flemish employees in a field-based approach.

**Methods:**

Employees (n=213) participated in a 3-group randomized controlled trial that assessed outcomes at baseline, 1-month follow-up, and 3-month follow-up through self-reports. A subsample (n=122) were willing to wear an activity monitor (activPAL) from Monday to Friday. The tailored group received an automated Web-based, computer-tailored intervention including personalized feedback and tips on how to reduce or interrupt workplace sitting. The generic group received an automated Web-based generic advice with tips. The control group was a wait-list control condition, initially receiving no intervention. Intervention effects were tested with repeated-measures multivariate analysis of variance.

**Results:**

The tailored intervention was successful in decreasing self-reported total workday sitting (time × group: *P*<.001), sitting at work (time × group: *P*<.001), and leisure time sitting (time × group: *P*=.03), and in increasing objectively measured breaks at work (time × group: *P*=.07); this was not the case in the other conditions. The changes in self-reported total nonworkday sitting, sitting during transport, television viewing, and personal computer use, objectively measured total sitting time, and sitting and standing time at work did not differ between conditions.

**Conclusions:**

Our results point out the significance of computer tailoring for sedentary behavior and its potential use in public health promotion, as the effects of the tailored condition were superior to the generic and control conditions.

**Trial Registration:**

Clinicaltrials.gov NCT02672215; http://clinicaltrials.gov/ct2/show/NCT02672215 (Archived by WebCite at http://www.webcitation.org/6glPFBLWv)

## Introduction

In modern societies, adults spend the majority of their waking time in sedentary behaviors, that is, activities in a sitting or reclining posture characterized by a low energy expenditure [1]. Working hours are probably the largest contributor to overall daily sitting time in adults. Flemish and Australian employees spent, respectively, about 71% and 77% of their working hours being sedentary [2-4]. Increasing evidence suggests that too much sitting is related to adverse health outcomes, independent of physical activity levels [5,6]. Both the total amount of sitting and the pattern of sitting (ie, prolonged uninterrupted periods) are associated with several adverse health effects in adults, such as obesity, metabolic syndrome, type 2 diabetes mellitus, some cancers, and all-cause and cardiovascular disease mortality [5-8]. Therefore, interventions to reduce (limit the amount of sitting) or interrupt (limit prolonged sitting bouts) sitting at work are warranted [9].

The evidence regarding the effectiveness of workplace interventions focusing on occupational sitting is growing [10,11]. Current workplace interventions are single- or multicomponent programs implementing individual or environmental strategies to influence workplace sitting. Examples of individual strategies are face-to-face behavioral change counselling sessions [12], workplace information sessions [13,14], tailored support for individual behavioral change through goal setting [13,14], and motivational interviewing [13,14]. Environmental strategies include the introduction of sit-stand workstations [15-17], portable pedal machines [18], and prompting software reminders [19,20]. Effects of these strategies are promising, with reductions in (mostly objectively measured) workplace sitting time (ranging from -28 to -262 minutes/8-hour workday) [12-14,18], and fewer (-4.6 bouts) and shorter (-5.6 minutes) sitting bouts [13].

A review of sit-stand workstations suggests that they can be effective in reducing occupational sitting time (the pooled effect size of 7 intervention studies was -77 minutes of sitting/8-hour workday), without compromising work performance [21]. However, these types of environmental interventions may not be feasible due to their high initial costs [22]. As a result, alternative interventions targeting workplace sitting are needed [9] in order to find out which other strategies are effective. In addition, most of the previous effectiveness studies were conducted in relatively small (maximum 62 participants) samples of employees or by low-quality evaluation methods [23]. This suggests that workplace intervention studies in larger samples are needed to increase our understanding of effective strategies to influence workplace sitting.

One popular public health promotion method that has been shown to successfully change a variety of health-related behaviors (dietary behaviors, alcohol consumption, smoking habits, and physical activity) is Web-based computer tailoring [24-26]. Computer-tailored interventions require participants to complete 1 or more brief assessment surveys. Then, based on participants’ answers, a computer program selects the relevant feedback messages from a database (through if-then algorithms), with the intention to provide information that is as personally relevant as possible [26]. Delivered thought the Internet, this approach has several advantages, including low cost, no limitations due to time or location, and a large reach, as the Internet now has 2 billion users worldwide [27]. In addition, interactive Web-based interventions have several benefits. First, they create the opportunity for ongoing contact with, and support to, their users [28,29]. Second, they apply tools that support self-regulatory skills, such as goal-setting activities, self-monitoring tools, action planning, skill-building activities, email reminders, and booster sessions [28,29]. To our knowledge, no workplace interventions targeting sitting have examined the effectiveness of Web-based computer-tailored program, and it seems worthwhile to investigate the effects of this type of workplace interventions.

We developed a theory-driven, Web-based, computer-tailored intervention to influence sitting at work and found it to be acceptable in terms of the assessment questioning, interestingness, length, credibility, and relevance of the advice [30]. In this study, we aimed to investigate the effects of this computer-tailored intervention to influence workplace sitting [30] on objectively measured sitting time, standing time, and breaks from sitting, as well as self-reported context-specific sitting. We compared the computer-tailored advice with generic advice and a no-advice control at 1-month and 3-month follow-ups. We hypothesized that both interventions would result in beneficial changes compared with the control condition, but that the effects of the computer-tailored advice would be significantly greater than the effects of generic advice.

## Methods

### Study Design and Sample

**Figure 1:**
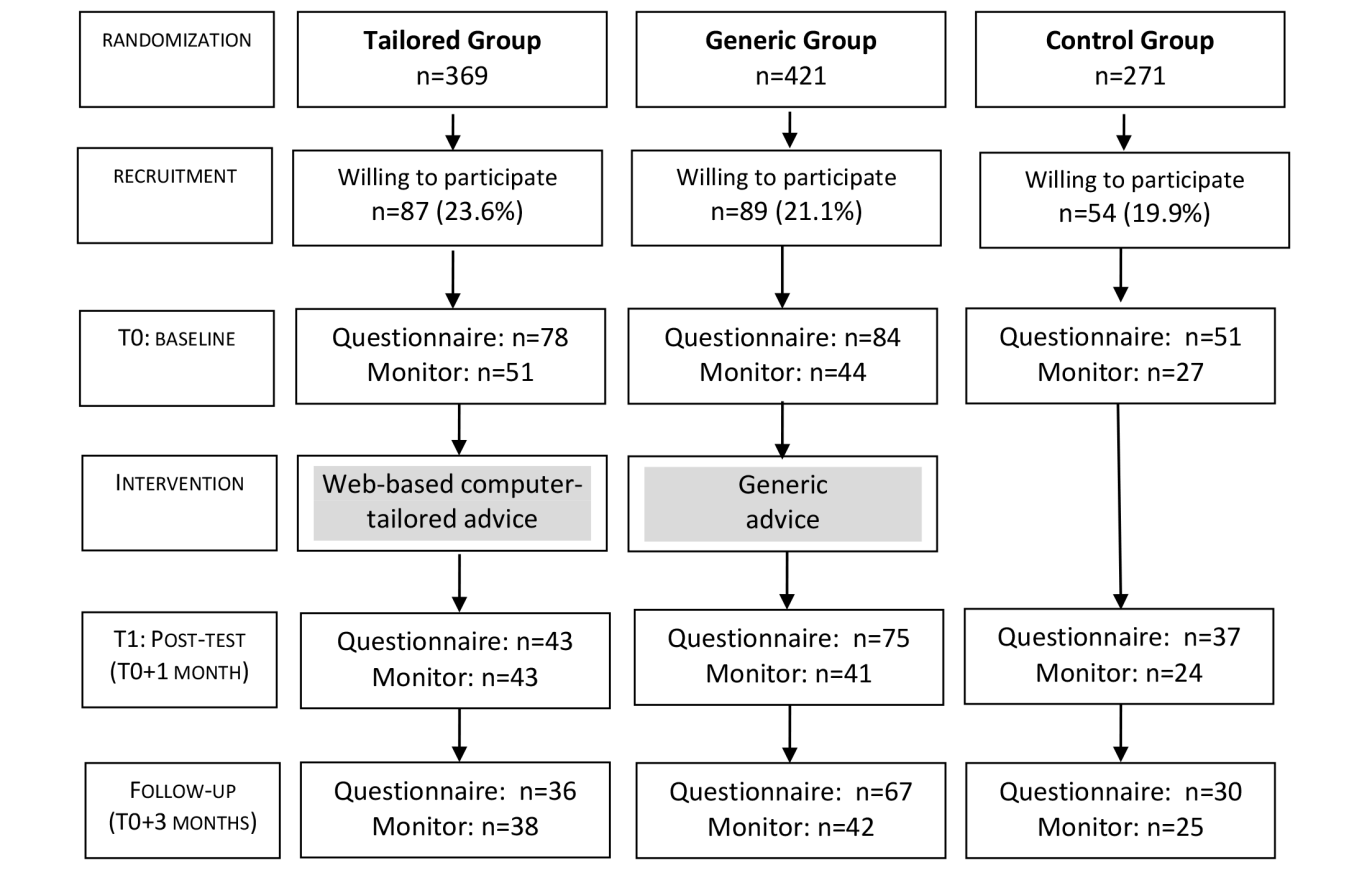
Flow chart of a randomized controlled trial of an intervention to reduce and interrupt sitting time at work.

The study used a controlled baseline (T0), 1-month follow-up (T1), and 3-month follow-up (T2: T0+3 months) design, with 3 different conditions (Figure 1 provides the study flow chart). The tailored group received a Web-based, computer-tailored intervention including personalized feedback and tips on reducing or interrupting workplace sitting (see the Interventions subsection below). The generic group received a Web-based intervention containing generic information and tips on reducing or interrupting workplace sitting. The control group was a wait-list control condition and received the generic intervention after completing all measurements.

We selected a convenience sample of 2 companies (a university and an environmental agency) in Flanders (ie, the northern, Dutch-speaking part of Belgium), mainly employing desk-based workers, having more than 100 staff members, and each having at least three different worksite locations. Both workplaces had a general health policy following European legislations and were informally committed to health aspects, but they did not yet focus on healthy sedentary behavior at work. We contacted company management by phone and email to inform them about the study, and both companies agreed to participate in this study (response rate 100%). Within the university, 3 departments of the central administration were selected to participate, and within the environmental agency, 3 departments in East Flanders were selected. Within each company, each department was randomly assigned to 1 of the 3 conditions. All selected departments were in different physical locations and the employees had little face-to-face contact with one another, reducing the opportunity for contamination between groups.

### Procedures

A contact person in each department provided the email addresses of all employees. In October 2014, employees were invited to participate by email. Employees willing to participate were asked to reply to the email within 1 week, indicating whether they wanted to complete the Web-based questionnaires only or whether they wanted to complete the Web-based questionnaires and additionally wear an activity monitor. We sent a reminder email with the invitation to those who had not yet responded, 1 day before the enrollment deadline. The study protocols were approved by the Ethics Committee of the Ghent University Hospital, Belgium.

A researcher emailed a confidential website username and password to all participants. After logging in, participants received a short introduction pop-up screen and were then referred to the home page, inviting them to complete an assessment questionnaire (see the Measures subsection below). After completing the baseline questionnaire, each group received different feedback. Those who were interested in participating but who did not complete the questionnaire within 7 days were sent up to 3 automatic reminders to visit the website.

A member of the research team visited the participants willing to wear an activity monitor at their workplace on a Monday. They were instructed to wear the monitor on the thigh from Monday to Friday. A researcher covered the monitor with a transparent medical tape (Tegaderm, 3M, Diegem, Belgium) before placement, and also attached the monitor itself with this tape. These waterproof attachments allowed for 24-hour wear, including water activities (eg, bathing and swimming). Monitor data from 3 days (Tuesday, Wednesday, and Thursday) were used for this study [31]. During the 3 full days of data collection, participants were also requested to complete a day log. On Friday, the researcher recollected the monitors at the workplace. These procedures were repeated at the 3 time points (T0, T1, and T2). The study was conducted between October 2014 and March 2015.

### Interventions

#### Computer Tailored Intervention

The development of this theory-driven intervention, called *Start to Stand*, has been described in detail elsewhere [30]. In brief, in this Web-based intervention, after completing an assessment questionnaire, users received personalized computer-tailored feedback about their sitting time, including tips and suggestions on how to interrupt (taking short standing breaks) and reduce (replacing sitting by periods of standing) sitting. Several questions on the Web-based questionnaire (see the Measures subsection below) were used to build up the feedback. The information requested from the participants referred to job-related information, knowledge about sedentary behavior, level of sitting time in 5 domains, frequency of interruptions in prolonged sitting, and level of physical activity. A set of decision rules selected the feedback messages that were matched and tailored to the specific answers given by the users. The combination of these feedback messages formed the tailored advice that appeared immediately on the user’s screen. We took into account constructs of the self-determination theory [32], the theory of planned behavior [33], and self-regulation theory [34]. Web-based interventions that use theory are more likely to have positive effects than those that are not theory based [35]. Interventions tailored to 4 or 5 theoretical concepts, such as social support, self-efficacy, and stages of change, were found to be more positive than interventions tailored to fewer than 3 theoretical concepts [36].

To increase their knowledge, participants first received general information about the importance of sitting behavior to improve health outcomes. This was followed by normative feedback about their own sitting behavior on working and nonworking days, in order to increase awareness of participants’ levels of sitting time. Further, feedback on the frequency of breaks from sitting, information on the importance of these breaks for health, and the suggestion to interrupt prolonged sitting every 30 minutes was provided [14,37]. Also the negative health impact of too much sitting, independent of physical activity levels, was explained. Next, feedback on participants’ physical activity level was given (see [30] for details).

At the end of this advice (section 1), participants were able to request up to 5 other noncommittal sections if they were interested. In line with the self-determination theory [32], users were able to choose which section of the advice they wanted to request additionally, instead of forcing users to complete all assessment questions at once and providing extensive advice containing all information. Leaving a choice for the users is assumed to increase autonomous motivation, which is more likely to lead to behavioral change [32]. The focus of these additional sections was on sitting during work hours (section 2: standing breaks; section 3: replacing sitting by standing), sitting during commuting to work (section 4), sitting during lunch breaks at work (section 5); and on making an action plan to improve sitting behavior (section 6). For each section, a brief questionnaire assessed participants’ current sitting behaviors and related psychosocial correlates (attitudes, self-efficacy, social support, intentions, and perceived benefits and barriers) [30]. Throughout the advice, the tailoring constructs were based on the theory of planned behavior [32], a theory focusing on the intention (or motivation) to adopt or modify a behavior and assuming that the intention is higher when people have more positive attitudes, perceived social influence, and self-efficacy. After participants completed each assessment questionnaire, the tailored advice appeared immediately on the computer screen. Participants were given personalized feedback about their attitudes, self-efficacy, social support, knowledge, intentions, and perceived benefits and barriers related to reducing or interrupting sitting.

In the last section, participants who were motivated to change their sitting were invited to create an action plan to convert intentions into specific actions through the specific, measurable, attainable, relevant, and time-bound (SMART) goals and implementation intentions [34,38], as goal setting has been found to be a common and effective intervention technique used in other health behavioral change programs. The action plan operates within the self-regulation theory, targeting pre- and postintentional processes to guide behavioral change [34]. Users were asked *what* (increase standing breaks, or replace sitting by standing, or both) they wanted to do, *how long*, *how often*, *when*, and *how* in order to state personally relevant and attainable goals. Finally, users were able to select precomposed if-then statements to make an if-then plan. Users were also able to formulate if-then statements themselves in an open-ended question format. When these questions were completed, a schematic overview of this personalized action plan was immediately provided on the screen [30].

Some screenshots of the tool are provided in Multimedia Appendix 1.

#### Generic Intervention

In the generic advice condition, after completing the baseline assessment questionnaire, users received generic information on the importance of reducing and interrupting sitting, and generic tips and suggestions on how to interrupt (taking short standing breaks, 6 tips) and reduce (replacing sitting by periods of standing, 8 tips) sitting during work hours, (lunch) breaks, and commuting (topics similar to the tailored group). While the information covered the same topics as in the tailored group, the generic group didn’t receive personalized advice or an action plan, and all information appeared on a single screen page.

### Measures

#### Web-Based Questionnaire

The questionnaire consisted of several parts, and all questions were asked at T0, T1, and T2, except for unchangeable variables, such as height.

##### Sociodemographics

The following sociodemographic variables were assessed: sex, age, highest educational degree with 5 options dichotomized into low (no diploma, elementary school, secondary school) and high (high school, university) education, height, and weight.

##### Work-Related Variables

We asked about the number of workdays per week (1–7), average daily amount of time (hours and minutes) spent at the workplace (open-ended), occupational status (blue collar, white collar, management), and employment duration (14 categories ranging from 1–6 months to 55–60 years).

##### Self-reported Behavioral Measures

We assessed the level of sitting time in 5 domains using the Workforce Sitting Questionnaire (WSQ) [39]. This questionnaire assessed time spent sitting on a workday and a nonworkday for the last 7 days while (1) travelling to and from places, (2) being at work, (3) watching television, (4) using a computer at home (not work related), and (5) doing other leisure activities. The WSQ has acceptable reliability (interclass correlation coefficient=.63) and validity against objectively accelerometer-measured sitting time (*r*=.34 to *r*=.45) [39]. Participants were also asked to report the average number of breaks from sitting they have on a regular workday [40].

The validated International Physical Activity Questionnaire (IPAQ) short version [41,42] assessed the number of days and duration of time spent in walking, moderate-intensity physical activity, and vigorous-intensity physical activity in the preceding week.

#### Activity Monitor and Day Log

Sedentary behavior was measured objectively using the activPAL (PAL Technologies, Glasgow, UK) activity monitor (weight 15 g, dimensions 53 × 35 × 7 mm). This inclinometer, distinguishing periods of sitting or lying from standing and assessing breaks from sitting, has been validated (correlation between activPAL and direct observation: *R*^2^=.94) in adults [43] and was recommended to assess sedentary behavior [43,44].

Participants were requested to complete a day log and record the type of day (workday at home, workday at the workplace, or nonworkday), time of getting up, the start and end time of the working day, and the time of going to sleep.

#### Website Usage Statistics

We collected the number of participants requesting the different sections of the advice from the website administration. Google Analytics provided data on website visiting time [45].

### Data Reduction

Data recorded by activPAL were reduced using PAL Technologies software (version 6.4.1). We calculated waking sitting time by subtracting sleep time reported in the day log from the total sitting time recorded by the activPAL device. The percentage of working time spent sitting was calculated as sitting time during work hours/work hours × 100. We used similar formulas to calculate percentage of working time spent standing and number of breaks per working hour. We calculated average values for T0, T1, and T2 from the mean scores of the 3 measurement days.

Within the 5 domains assessed using the WSQ, we truncated values over 12 hours/day to 12 hours to avoid unrealistic values [30]. We calculated total time spent sitting on a workday and on a nonworkday by summing the time reported in every domain. These totals were truncated at 16 hours/day [30].

Based on the guidelines for data processing and analysis of the IPAQ [46], total scores for walking, and moderate and vigorous physical activities were computed (number of days × time duration). Body mass index (BMI) was calculated as weight in kg divided by height in m^2^.

### Data Analyses

All analyses were conducted in SPSS version 22.0 (IBM Corporation) and significance was set at *P*<.05, while *P* values between .05 and .10 were interpreted as indicating borderline significance. We did not perform any multilevel analyses because there were fewer than 10 clusters and none of the variables were measured at cluster level [47]. The 3 groups’ characteristics at baseline were compared using 1-way analysis of variance (for the quantitative variables age and BMI) or χ^2^ tests (for the qualitative variables sex, education, work regimen, occupational status, and employment duration). We conducted the same analyses to compare the characteristics of participants willing to wear the activity monitor at baseline and those not willing to wear it. Descriptive statistics for usage of the various website components are provided.

To investigate the 1-month and 3-month follow-up effects of the intervention, we conducted 3 repeated-measures multivariate analysis of variance tests with time (T0, T1, or T2) as the within-participants factor, condition (3 groups) as the between-participants factor, and self-reported sitting (workday and nonworkday total sitting; average daily domain-specific sitting) and objectively measured sitting (total waking sitting time, working time spent sitting, working time spent standing, breaks from sitting per work hour) as the dependent variables. When the time (3 levels) × condition (3 levels) effects were significant, we conducted additional post hoc repeated-measures analyses, including 2 times points (T0–T1 or T0–T2) and only 2 conditions, to find out where the differences in changes over time between the conditions occurred. We included the following covariates in the analyses: age, sex, education, hours at work, employment duration, BMI, walking, and moderate and vigorous-intensity physical activity at baseline. Due to the skewed nature of the outcomes, we did the analyses on square root transformations to improve normality, but for reasons of clarity, we report nontransformed average scores in the tables. We executed this approach using both a retained sample analysis (ie, completer analysis) and an intent-to-treat analysis (last value carried forward). Because we found no differences between the 2 analyses, we report results only of the retained sample analysis.

## Results

### Participants’ Characteristics at Baseline and Website Usage

The total sample (N=213) of employees completing the Web-based questionnaire consisted of 31.5% (67/213) men, 81.7% (174/213) with a high level of education, 91.5% (195/213) who were white collar workers, and 69.5% (148/213) with an employment duration of more than 5 years. Participants had a mean age of 40.3 (SD 9.1) years, worked on average 8.0 (SD 0.7) hours/day, and had a mean BMI of 23.9 (SD 3.4) kg/m^2^. Table 1 gives an overview of the baseline variables for the 3 intervention groups. The 3 groups did not differ in sociodemographic, work-related, and health-related variables (see Table 1). Self-reported and objectively sitting variables at baseline are presented below. Of those completing the questionnaires, 122/213 (57.3%) employees were willing to wear the activity monitor at baseline. There were no differences between participants who were willing and those who were not willing to wear the activity monitor across all variables (sociodemographic, work-related, health-related, and self-reported sitting time variables; data not shown).

**Table 1:** Baseline characteristics for the 3 study groups.

Variables		Tailored group (n=78)	Generic group (n=84)	Control group (n=51)	Group comparisons	
					*F*_df_or χ^2^_df_	*P* value
Sociodemographic variables						
	Age in years, mean (SD)	40.5 (8.6)	40.7 (9.7)	39.3 (9.0)	*F*_2,209_=0.44	.65
	Males, n (%)	25 (32.1)	27 (32.1)	15 (29.4)	χ^2^_2,212_=1.9	.76
	High school/university education: n (%)	58 (75.3)	70 (83.3)	46 (90.2)	χ^2^_2,212_=4.8	.09
Work-related variables						
	Hours at work, mean (SD)	8.0 (0.9)	8.0 (0.6)	8.0 (0.6)	*F*_2,211_=0.36	.70
	White collar occupational status, n (%)	74 (96.1)	75 (89.3)	46 (90.2)	χ^2^_2,212_=2.8	.24
	Employment duration>5 years, n (%)	55 (71.4)	56 (66.7)	37 (72.5)	χ^2^_2,212_=0.7	.72
Health-related variables						
	BMI^a^in kg/m^2^, mean (SD)	24.2 (3.1)	23.6 (3.5)	23.7 (3.5)	*F*_2,211_=0.75	.48
	Walking time in minutes/day, mean (SD)	18.8 (28.3)	21.1 (21.6)	18.6 (19.0)	*F*_2,208_=0.30	.74
	Moderate-intensity PA^b^in minutes/day, mean (SD)	24.7 (26.9)	19.3 (20.1)	18.0 (19.0)	*F*_2,208_=1.69	.19
	Vigorous-intensity PA in minutes/day, mean (SD)	8.4 (11.5)	11.6 (15.6)	9.9 (15.6)	*F*_2,209_=1.05	.35

^a^BMI: body mass index.

^b^PA: physical activity.

At baseline, all 78 participants in the tailored group completed section 1 (100%). The average time needed to complete the assessment survey was 16.3 minutes. Time spent on the first advice page was on average 20.1 minutes. A total of 66/78 participants completed section 2 (84.6%), 64/78 completed section 3 (82.1%), 60/78 completed section 4 (76.9%), 59/78 completed section 5 (75.6%), and 54/78 completed an action plan (69.2%).

### Intervention Effects on Self-reported Sitting Measures

Table 2 presents the baseline, 1-month, and 3-month follow-up values of the self-reported sitting for each group. Total workday sitting change from baseline to follow-up was significantly different between the 3 groups (see Table 2). The decrease in sitting time in the tailored group was significantly greater than the decrease in the generic group (*P*=.002) and the increase in the control group (*P*=.002). The decrease in the generic group was borderline significantly different from the change in the control group (*P*=.05). The changes over time in total nonworkday sitting did not differ significantly between the groups (see Table 2).

**Table 2:** Mean self-reported sitting at baseline (T0), 1-month follow-up (T1), and 3-month follow-up (T2) for the 3 groups and time × group effects.

Group			T0	T1	T2	*F*_dftime×group_	*P* value
Total sitting in minutes/day, mean (SD)							
	Total workday sitting					T0–T1–T2: *F*_4,128_=5.65	<.001***
		Tailored (n=36)	507 (104)	480 (128)	425 (110)	T0–T1: *F*_2,149_=1.45	.24
		Generic (n=64)	457 (107)	444 (105)	437 (95)	T0–T2: *F*_2,128_=8.47	<.001***
		Control (n=28)	449 (126)	434 (131)	469 (92)		
	Total nonworkday sitting					T0–T1–T2: *F*_4,128_=1.20	.31
		Tailored (n=36)	141 (70)	139 (69)	132 (70)	T0–T1: *F*_2,149_=0.63	.54
		Generic (n=64)	130 (63)	131 (67)	141 (77)	T0–T2: *F*_2,128_=1.15	.32
		Control (n=28)	123 (58)	117 (47)	134 (55)		
Domain-specific sitting in minutes/day, mean (SD)							
	Sitting at work^a^					T0–T1–T2: *F*_4,118_=6.72	<.001***
		Tailored (n=33)	338 (107)	279 (92)	259 (88)	T0–T1: *F*_2,138_=12.5	<.001***
		Generic (n=61)	288 (59)	279 (64)	280 (69)	T0–T2: *F*_2,119_=10.09	<.001***
		Control (n=24)	281 (65)	280 (50)	288 (48)		
	Sitting during transport^b^					T0–T1–T2: *F*_4,118_=0.45	.77
		Tailored (n=33)	78 (84)	103 (124)	58 (49)	T0–T1: *F*_2,138_=0.47	.63
		Generic (n=61)	66 (79)	60 (67)	48 (31)	T0–T2: *F*_2,119_=0.01	.98
		Control (n=24)	81 (106)	74 (88)	62 (62)		
	Television viewing^b^					T0–T1–T2: *F*_4,118_=1.31	.23
		Tailored (n=33)	100 (57)	104 (56)	106 (61)	T0–T1: *F*_2,138_=1.24 0.29	.29	
		Generic (n=61)	95 (62)	92 (67)	102 (68)	T0–T2: *F*_2,119_=1.24	.29
		Control (n=24)	91 (68)	79 (68)	82 (61)	
	Personal computer use^b^					T0–T1–T2: *F*_4,118_=1.51	.20
		Tailored (n=33)	50 (46)	53 (47)	47 (29)	T0–T1: *F*_2,138_=0.20	.82
		Generic (n=61)	51 (40)	52 (44)	51 (39)	T0–T2: *F*_2,119_=1.35	.26
		Control (n=24)	58 (62)	59 (71)	69 (65)		
	Other leisure time sitting^b^					T0–T1–T2: *F*_4,118_=1.86	.12
		Tailored (n=33)	101 (42)	90 (44)	75 (32)	T0–T1: *F*_2,138_=1.68	.19
		Generic (n=61)	99 (61)	98 (61)	97 (46)	T0–T2: *F*_2,119_=3.64	.03*
		Control (n=24)	95 (48)	96 (43)	102 (64)		

^a^Average on workday.

^b^Average of workday and nonworkday.

* *P*<.05; *** *P*<.001.

Analyses of the domain-specific sitting data showed that changes over time in sitting at work and other leisure time sitting differed significantly between the 3 groups. There was a decrease in sitting at work in the tailored group, which was significantly greater than the changes in the generic group (T0–T1: *P*<.001, T0–T2: *P*<.001) and the control group (T0–T1: *P*=.001, T0–T2: *P*=.001). The changes over time did not differ significantly between the generic group and the control group (T0–T1: *P*=.48, T0–T2: *P*=.26). There was also a decrease in other leisure time sitting from baseline to follow-up in the tailored group, which was significantly greater than the changes over time in the generic group (*P*=.007) and the control group (*P*=.02). The changes from baseline to follow-up did not differ significantly between the generic group and the control group (*P*=.78). The changes over time in sitting during transport, television viewing, and computer use did not differ significantly between the 3 groups (see Table 2).

### Intervention Effects on the Objectively Measured Variables

Table 3 gives an overview of the sedentary behavior measures derived from the activPAL at baseline, and 1-month and 3-month follow-ups for each group. The changes over time in total sitting while being awake, sitting at work, and standing at work did not differ significantly between the 3 groups (see Table 3). For breaks at work, the only borderline significant difference was found between the tailored group (slight increase in breaks from baseline to follow-up) and the generic group (slight decrease in sitting time from baseline to follow-up, *P*=.07).

**Table 3:** Mean objectively measured variables at baseline (T0), 1-month follow-up (T1), and 3-month follow-up (T2) for the 3 groups and time × group effects.

Group		T0	T1	T2	*F*_dftime x group_	*P* value
Total sitting time awake in hours/day, mean (SD)					T0–T1–T2: *F*_4,75_=0.56	.69
	Tailored (n=35)	576 (109)	600 (91)	607 (117)	T0–T1: *F*_2,80_=0.52	.60
	Generic (n=35)	578 (101)	574 (103)	576 (109)	T0–T2: *F*_2,79_=1.06	.35
	Control (n=23)	605 (96)	616 (115)	623 (100)		
Sitting at work in % work hours, mean (SD)					T0–T1–T2: *F*_4,75_= *F*_4,75_=0.22	.93
	Tailored (n=35)	66.8 (15.5)	71.7 (14.0)	69.0 (13.7)	T0–T1: *F*_2,80_=0.28	.76
	Generic (n=35)	69.0 (13.8)	71.2 (15.1)	68.8 (15.1)	T0–T2: *F*_2,79_=0.12	.89
	Control (n=23)	74.3 (15.5)	78.3 (11.1)	74.8 (13.5)		
Standing at work in % work hours, mean (SD)					T0–T1–T2: *F*_4,75_=0.10	.98
	Tailored (n=35)	24.7 (13.5)	22.2 (9.0)	23.6 (11.7)	T0–T1: *F*_2,80_=0.05	.95
	Generic (n=35)	24.4 (11.3)	22.7 (15.4)	24.3 (14.4)	T0–T2: *F*_2,79_=0.11	.90
	Control (n=23)	16.3 (9.3)	17.1 (7.9)	17.8 (9.0)		
Breaks at work in no/work hour, mean (SD)					T0–T1–T2: *F*_4,75_=2.54	.09*
	Tailored (n=35)	3.8 (1.5)	3.7 (1.3)	4.3 (1.6)	T0–T1: *F*_2,80_=0.72	.40
	Generic (n=35)	3.6 (1.3)	3.6 (1.4)	3.5 (1.3)	T0–T2: *F*_2,79_=2.40	.11
	Control (n=23)	3.0 (1.4)	3.2 (1.4)	3.3 (1.6)		

* *P*<.10

## Discussion

To our knowledge, this is the first randomized controlled study evaluating 1-month and 3-month follow-up effects of a theory-driven, Web-based, computer-tailored intervention to reduce or interrupt sitting among employees. Results are promising, with positive intervention effects on self-reported sitting time at work, self-reported sitting time during leisure, and objectively measured breaks at work. For these outcomes, the tailored intervention had superior effects to those of the control and the generic condition, confirming our hypothesis. This suggests the significance of computer tailoring in targeting sedentary behavior, as also seen in Web-based advice for other health-related behaviors, such as physical activity and diet [24-26]. However, the expectation that the effect of the generic condition would differ from the control condition was not confirmed. The provision of nontailored Web-based advice seems not sufficient to result in sedentary behavioral change, which is in contrast to behavioral change interventions targeting physical activity or dietary habits, in which generic interventions did have positive effects [48,49].

It should be noted that the positive findings concerning the decrease in self-reported sitting duration were not reflected in the objective measures, as no effect was found on activPAL-measured total sitting time or sitting time at work. This result emphasizes the importance of combining self-reported and objective measures. The effectiveness study of sit-stand workstations conducted by Chau et al [50] found the opposite pattern, namely positive effects for sitting time at work measured by the activPAL, but no effects for sitting time at work measured with the WSQ. The review of Prince et al [9] found no significant effect differences between self-reported and objectively measured sedentary time. It should be noted that most studies of the review used one of two types of measures: either objective or self-reported measures, but not both. When looking for potential explanations for the discrepancy between our self-reported and objectively measured data, we conducted post hoc correlation analyses (*r*=.07 for total sitting and *r*=.11 for work-related sitting), which suggested that the perception of self-reported sitting was different from objectively measured sitting. Some other studies showed that participants underestimated their self-reported sedentary behavior [51]. In contrast, other validation studies of the WSQ, using accelerometers, showed correlates of .34 and more [39]. Based on our study and earlier studies [51], the combination of self-reported and objective measurements is recommended to fully explore findings. A second possible explanation may be that the sample of participants providing the activPAL data were only a subsample (122/213, 57.3% of the total sample at baseline, 105/133, 78.9% at 3-month follow-up). Still, intervention effects on the self-reported measures were not different between employees wearing the monitor and those who did not (data not shown). Even though the subsample is quite large for a field-based study, objective data should ideally have been available for all participants, as the observed statistical power was low to find differences between groups in total sitting and in sitting at work (observed power ranged from .12 to .36) and in standing time at work (observed power=.06).

Compared with baseline, the self-reported work-related sitting time in the tailored condition was lower at 1-month follow-up (-59 minutes/day) and 3-month follow-up (-79 minutes/day), while this was not the case for the other conditions. These reductions in sitting time are similar to those seen in interventions implementing activity-permissive workstations (-77 minutes/8-hour workday) as shown in the review of Neuhaus et al [21], which included 38 studies. Our reductions in sitting were even higher than those found in a meta-analysis of 34 lifestyle intervention studies (including 8 workplace interventions) having sedentary behavior as an outcome, showing an overall reduction in sedentary time by mean differences of -22 minutes/day in favor of the intervention group [52]. A reduction in sedentary time of just 30 minutes/day is suggested to have clinically meaningful effects on health [9], which indicates that our computer-tailored intervention may potentially be useful in public health promotion. However, in the review of Martin et al [53], the authors concluded that it is not known whether the effective interventions aimed at reducing sedentary behavior resulted in clinically meaningful and sustained improvements in health outcomes, as it was not possible to determine the intervention effect of reduced sedentary behavior on cardiometabolic risk, body composition, and mental health outcomes. Still, in a Spanish Internet-delivered workplace intervention focusing on decreasing occupational sitting (via goal setting for step counts and walking), occupational sitting time (-22 minutes/day) and waist circumference (-0.8 cm) decreased in the intervention group [54].

In our subsample wearing the activPAL, we found a positive intervention effect at follow-up for breaks during work (+0.4 breaks/work hour, ~3.2 breaks in an 8-hour workday, an increase from 30 to 34 breaks in an 8-hour workday) in the tailored condition compared with no change in the generic condition. In the review of Martin et al [53], pooled intervention effects on sedentary behavior patterns indicated no statistically significant effect for the number of sitting breaks per hour. The clinical meaning of the effect we observed is, however, unknown, as the dose-response relation regarding breaks is unclear [52]. Still, an eHealth intervention to reduce prolonged occupational sitting that passively prompted desk-based employees every 45 minutes to stand and perform nonexercise physical activity did examine the clinical effects. The study resulted in activity breaks about 6 times/workday for about 1.3 minutes at a time in the experimental group and showed that the mean arterial pressure significantly decreased [55] and the work-related energy expenditure increased [56] in the experimental group compared with a control group. Given that we found an increase in breaks in our study, we can assume that this computer-tailored intervention can also result in significant health effects; however, we did not track the duration of the activity breaks.

It may be surprising that an individual-based intervention such as our computer-tailored intervention resulted in such a relatively high reduction in self-reported sitting time at work. In the case of implementing sit-stand workstations, it is reasonable that sitting time is substantially reduced, as the environment is changed to do so, but without standing desks one could expect the intervention to have less effect on total sitting duration and more on the sitting pattern. Based on the feasibility and acceptability study of our computer-tailored intervention, pointing out that employees perceived interrupting sitting to be more achievable than reducing workplace sitting, this could also be expected to be the case here [30]. Nevertheless, our intervention seemed to affect both the pattern (objectively measured data) and the duration (self-reported data) of sitting.

We found no other 1-month follow-up effects on self-reported outcomes (sitting during transport, television viewing, computer use, leisure time sitting) or other objectively measured outcomes (total sitting time, sitting and standing time at work). However, we did find significant and positive 3-month follow-up intervention effects for self-reported total workday sitting and self-reported leisure time sitting. The change over time was more positive in the tailored condition than in the other conditions. The fact that leisure time sitting decreased (-26 minutes/day) from baseline to follow-up was surprising, as the advice mostly focused on work-related aspects: work hours, commuting, and (lunch) breaks. Still, this may mean that employees transferred the information and tips regarding one specific setting (work) to another (leisure). The study of Chau et al [50] obtained a similar result, showing that self-reported television viewing time decreased after a workplace intervention in which employees used a sit-stand workstation for 4 weeks. The authors argued that a reduction in television viewing time would be a welcome side effect of their intervention, especially because compensatory effects for occupational sitting (ie, less sitting at work would lead to more sitting at home) were not found in other previous studies [57-59]. In our study, however, we found no effect on television viewing.

### Study Strengths and Limitations

Our study has several strengths and limitations to take into account. The first strength is the randomized controlled design with a large sample of employees relative to other workplace interventions focusing on sedentary behavior. Second, the use of the activPAL as an objective measure for the outcomes was a strength, as self-reported measures can have recall and social desirability biases. However, as stated earlier, the first limitation is that we used this monitor only in a subsample of employees willing to wear the monitor, which is probably a result of the field-based approach of this study. Further, from a methodological point of view, it would have been more suitable to randomly allocate the monitors within the total sample in order to avoid sampling bias. However, from a compliance point of view, we believed it was better to provide a monitor only to those willing to wear one, in order to limit dropout. The dropout rate was lower (17/122, 13.9%) in the group wearing a monitor (and completing the questionnaires) than in the sample only completing the questionnaires (80/213, 37.6%). A study on the retention rates in physical activity interventions in workplace, health care, and home- or community-based settings revealed a mean retention rate of 78%, with minimal differences between intervention settings [60]. In our study, the mean retention rate was 74.3% (only questionnaire: 133/213, 62.4%; questionnaire and monitor: 105/122, 86.1%). The second limitation, probably resulting from the field-based approach, is the relatively low initial response rate (230/1061, 21.5%), which is comparable with another workplace intervention study using only 1 mailing to recruit (response rate 20%) [61]. The study of Waters et al [60] showed that response rates in physical activity interventions in adults range from 20% to 89%. Third, we recruited participants in only 2 companies, with worksites in 3 different settings, probably resulting in different workplace cultures. In addition, the study sample consisted of mainly healthy weight, highly educated women. This is in line with other workplace physical activity interventions in which participants were found to be younger, more educated, and healthier than nonparticipants [60]. All these factors may compromise generalizability of the results. Fourth, we do not know the long-term effects of this intervention, as we completed the follow-up measures only 3 months after baseline. Martin et al [53] found that interventions of up to 3 months resulted in a significant reduction in sedentary time (-48 minutes/day) in favor of the intervention group, whereas longer intervention durations of more than 3 months did not show beneficial intervention effects.

This study opens perspectives for future research. The effect of this intervention on psychosocial correlates should be tested, including the mediating effect of the change in these factors on the behavioral effects. As it stands, we do not know what the active intervention components are. Further, future research should investigate whether this tailored intervention would be more effective in combination with other (environmental) strategies, for example the use of sit-stand desks. Previous interventions also chose multicomponent programs to tackle the problems of too much sitting [50]. Therefore, this intervention could be an additional component in studies based on ecological models, intervening simultaneously at multiple levels.

### Conclusions

To our knowledge, this is the first intervention study to describe the effectiveness of a theory-driven, Web-based, interactive computer-tailored intervention aimed at reducing and interrupting sitting at work. The computer-tailored approach showed promising outcomes to address sitting time, as the tailored intervention was successful in decreasing self-reported sitting time at work and during leisure time, and in increasing objectively measured breaks at work compared with the generic and control conditions, which had no significant impact. This suggests that this computer-tailored intervention might have potential to contribute to the health promotion field.

## Appendix

Multimedia Appendix 1 Screenshots of the start to stand tool.

Multimedia Appendix 2 CONSORT-EHEALTH (V 1.6.1) checklist.

## Abbreviations

BMI: body mass index
IPAQ: International Physical Activity Questionnaire
SMART: specific, measurable, attainable, relevant, and time-bound
T0: baseline
T1: 1-month follow-up
T2: 3-month follow-up
WSQ: Workforce Sitting Questionnaire
