# Supplementary material for: The Effectiveness of a Web-Based Computer-Tailored Intervention on Workplace Sitting: A Randomized Controlled Trial
Source: J Med Internet Res. 2016 May 31;18(5):e96. doi: 10.2196/jmir.5266 (PMC4908304; doi:10.2196/jmir.5266)

Screen shot log-in page:

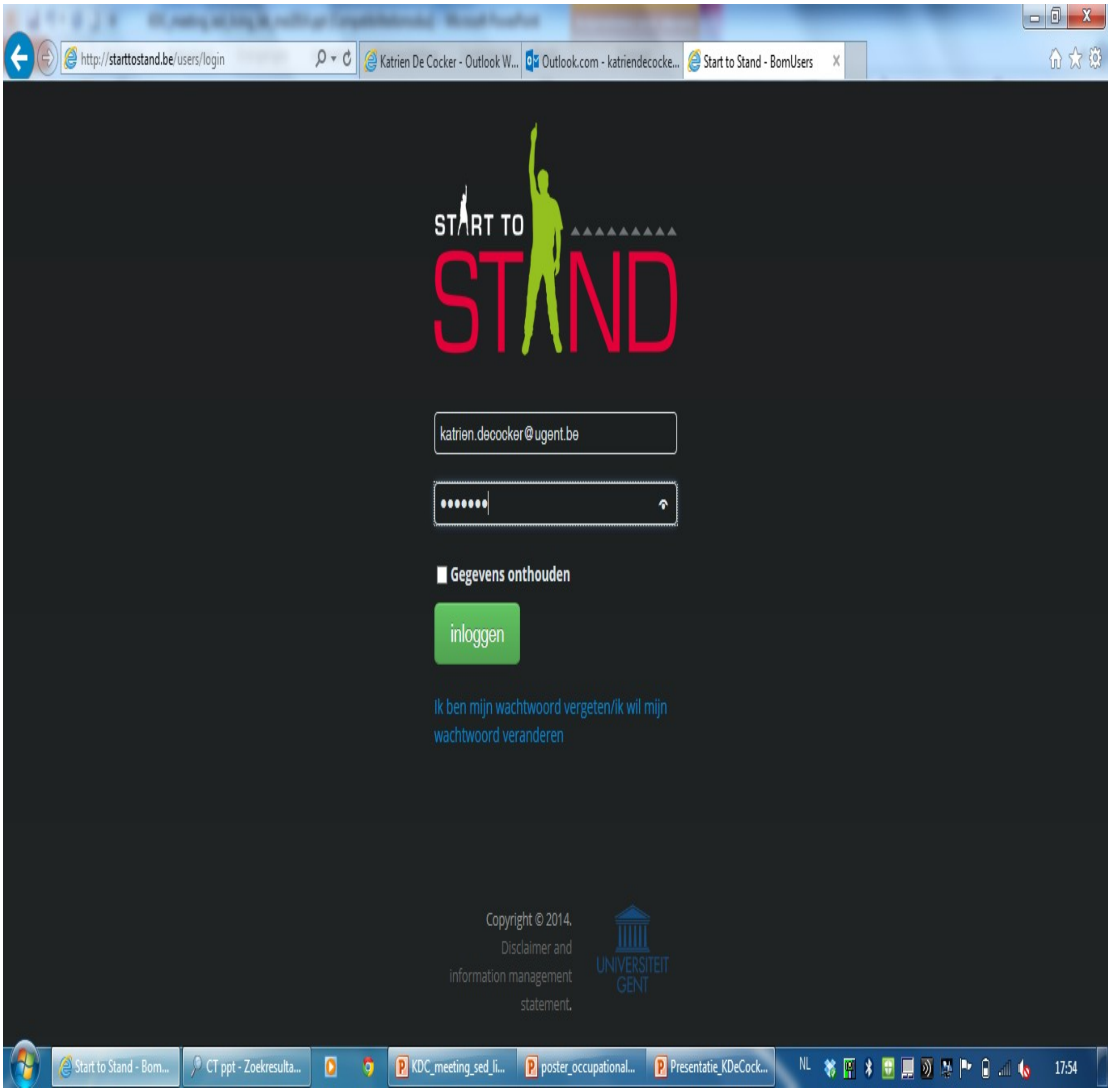

Screen shot assessment questionnaire section 2:

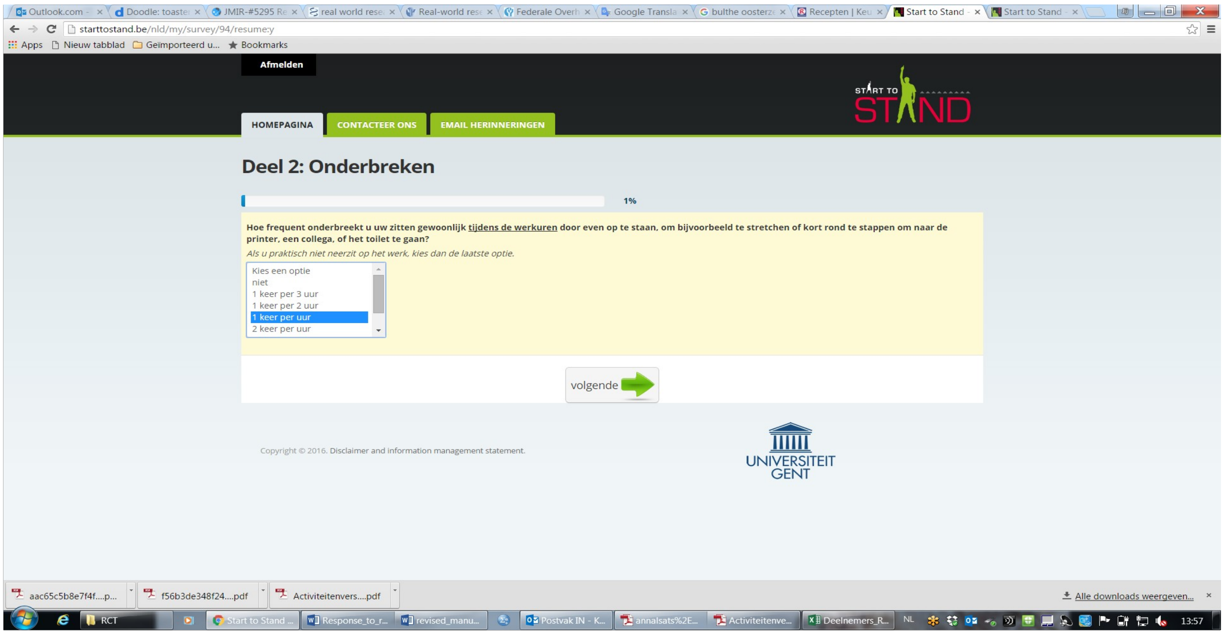

Screen shot assessment questionnaire action planning:

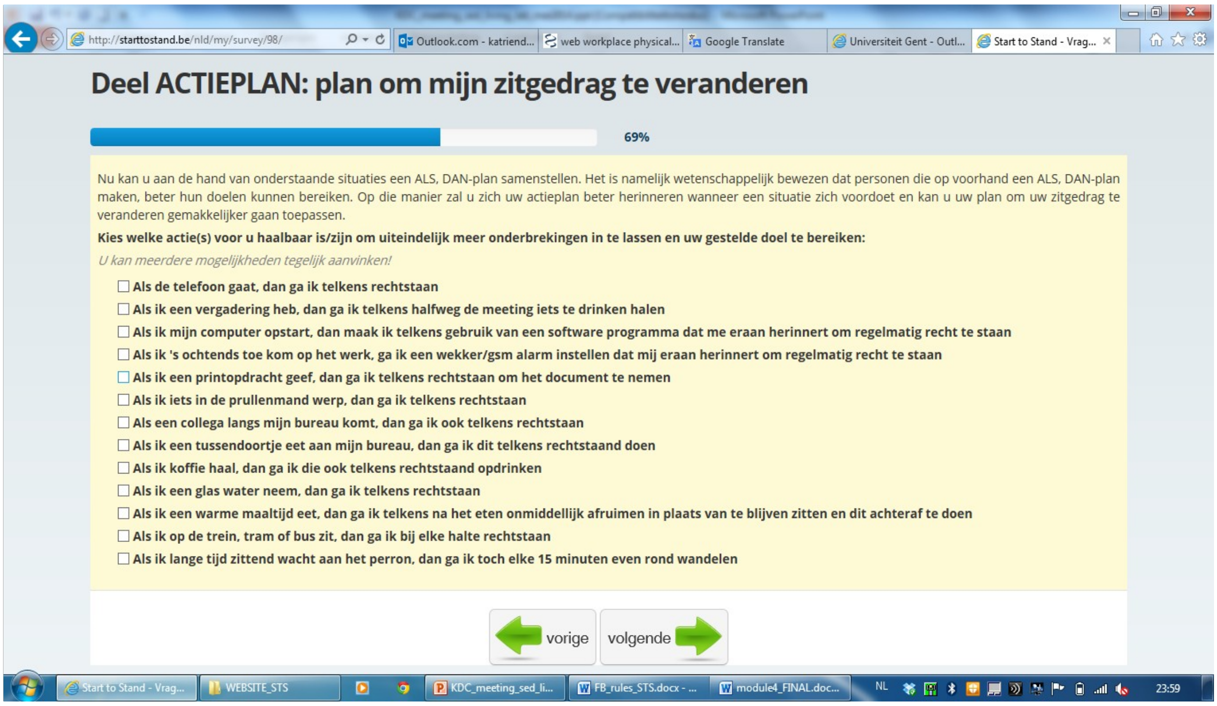

Screen shot advice page section 1 :

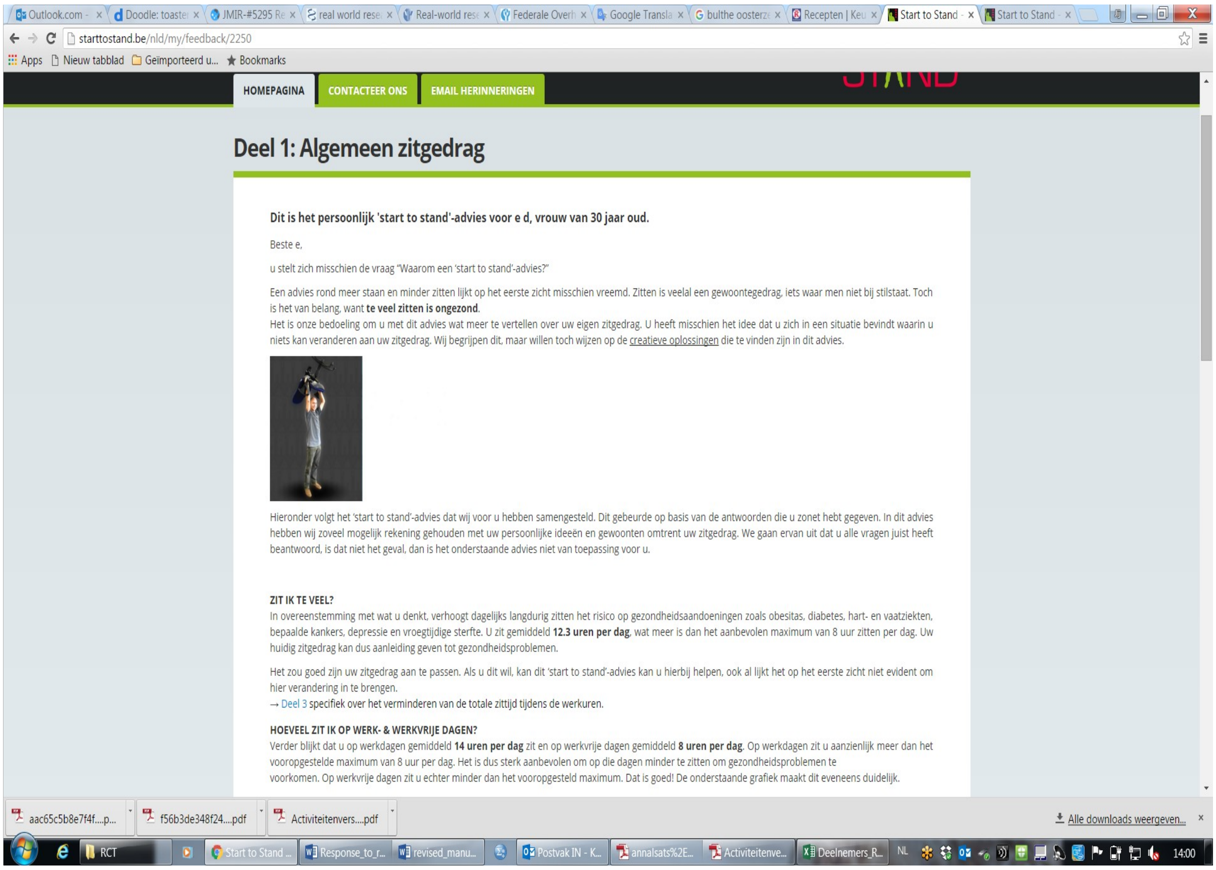

Screen shot advice page action planning :

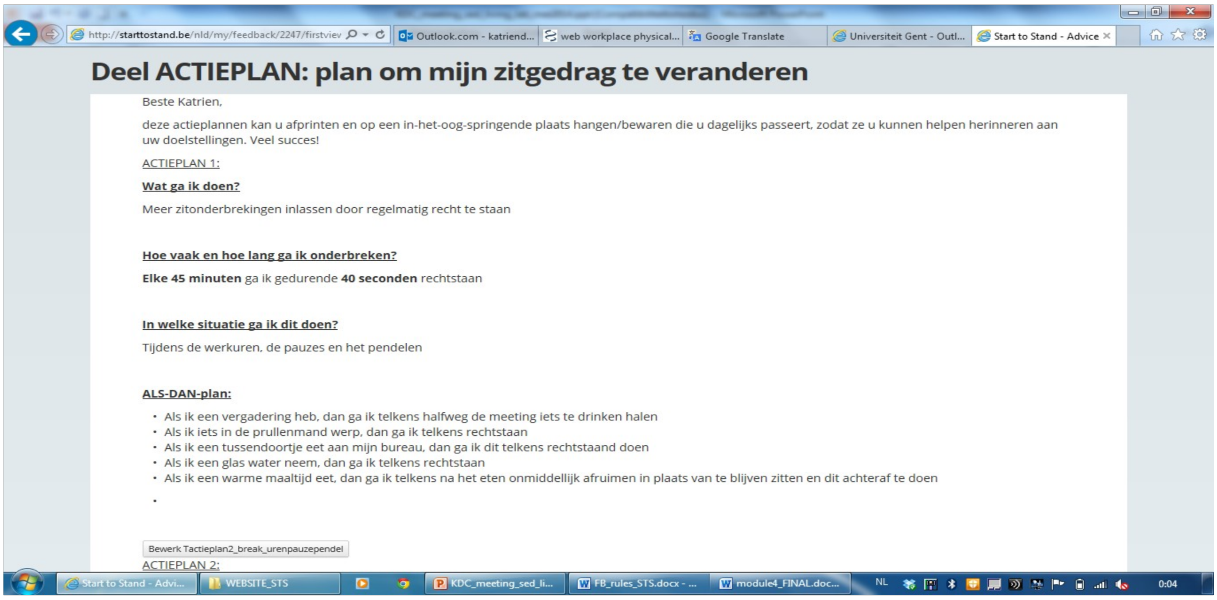

Supplement: Multimedia Appendix 1 [file jmir_v18i5e96_app1.pdf]
